# Supplementary material for: Were changes in stress state responsible for the 2019 Ridgecrest, California, earthquakes?
Source: Nat Commun. 2020 Jun 17;11:3082. doi: 10.1038/s41467-020-16867-5 (PMC7299982; doi:10.1038/s41467-020-16867-5)
Supplement: Supplementary file 2 — Supplementary Information [file 41467_2020_16867_MOESM2_ESM.pdf]

Supplementary Information

Were changes in stress state responsible for the 2019 Ridgecrest, California, earthquakes?

K. Z. Nanjo

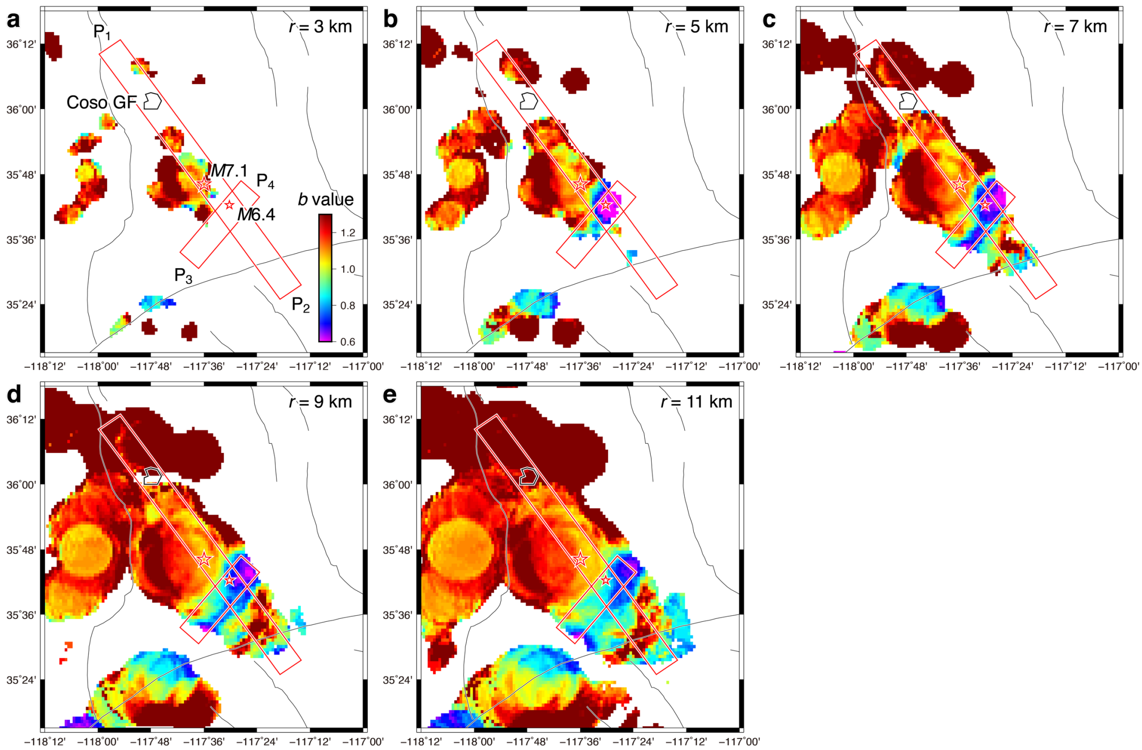

Supplementary Figure 1. Pre-*M*6.4-quake *b*-values. Distribution of *b*-values was obtained from seismicity ($M \geq 1$ and depth of 7-13 km) during the period from 1980 to immediately before the *M*6.4 quake, using different radii (*r*): (**a**) 3 km, (**b**) 5 km, (**c**) 7 km, (**d**) 9 km, and (**e**) 11 km. **b** is the same as Fig. 1**b**.

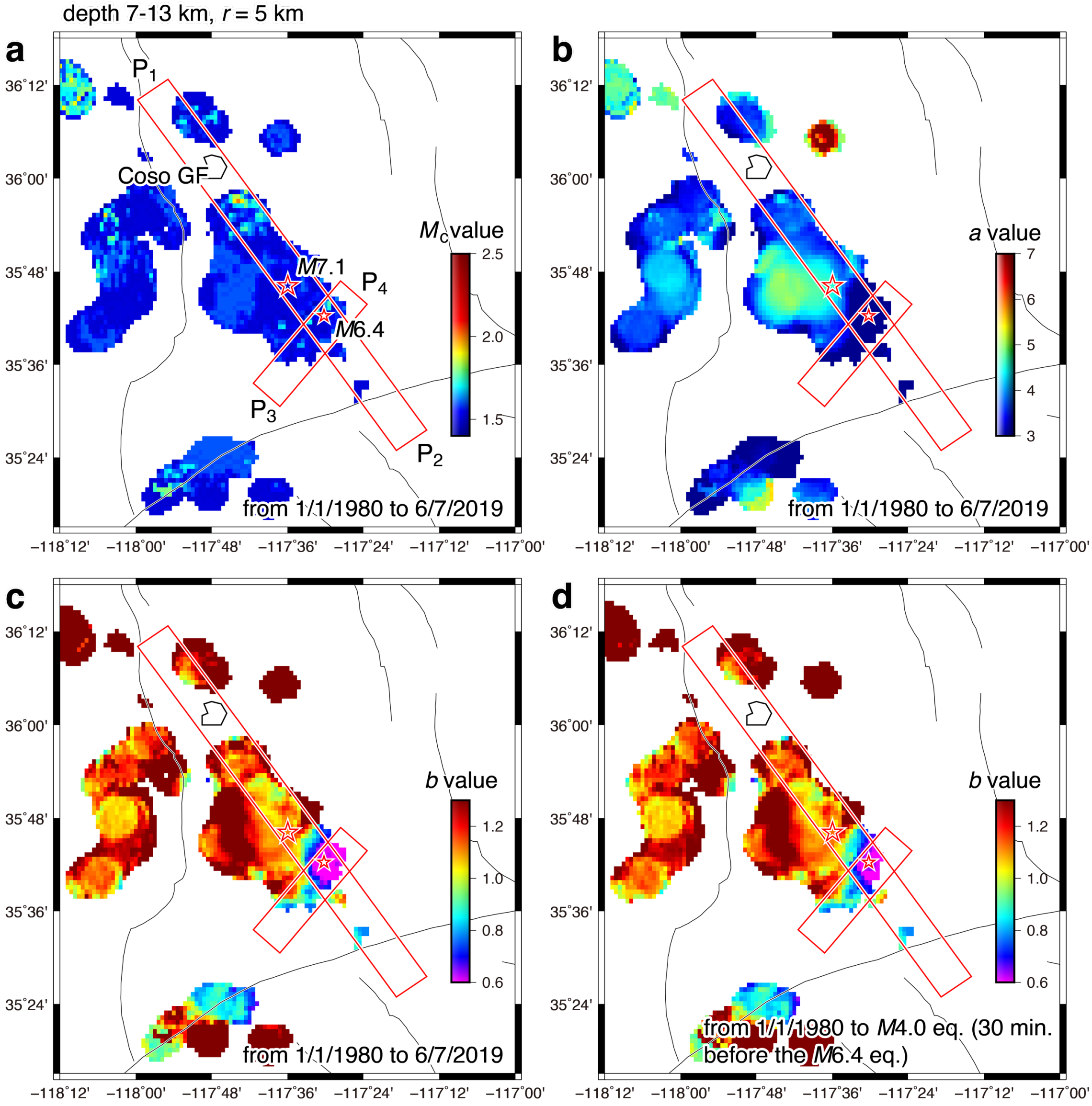

Supplementary Figure 2. Maps of M_c-, *a*-, and *b*-values. (**a**) Map of M_c obtained from seismicity ($M \geq 1$ and depth of 7-13 km) during the period from 1980 to immediately before the *M*6.4 quake. (**b**) Same as **a** for *a*-values. (**c**) Same as **a** for *b*-values. This is the same as Fig. 1**b**. (**d**) Same as **c** for seismicity during the period from 1980 to immediately before the *M*4.0 quake that occurred about 30 minutes before the *M*6.4 quake near the eventual *M*6.4 hypocenter.

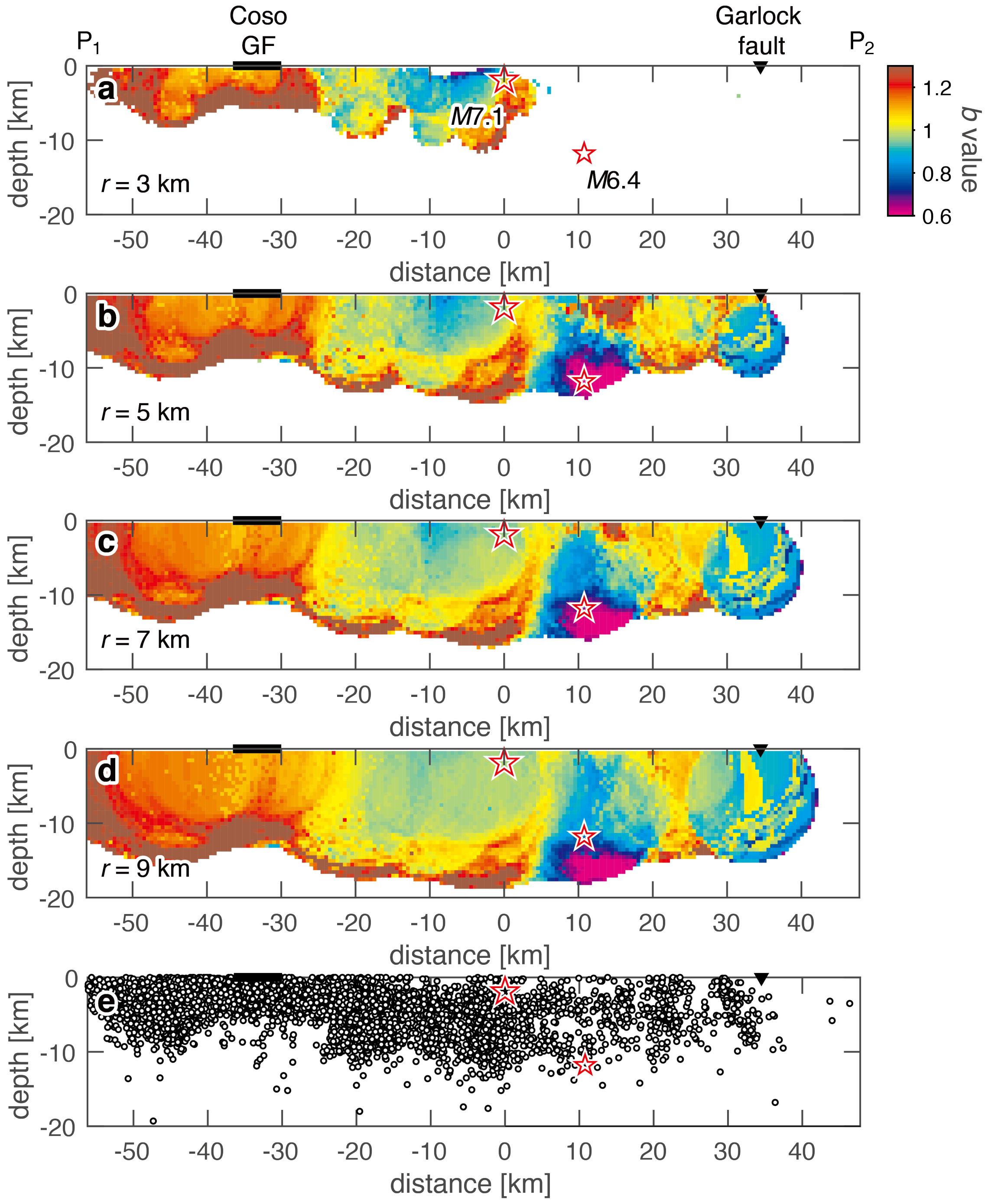

Supplementary Figure 3. Distribution of *b*-values along the fault ruptured by the *M*7.3 quake. This was computed with the NCSN catalog from 1980 to immediately before the *M*6.4 quake, using different radii (*r*): (**a**) 3 km, (**b**) 5 km, (**c**) 7 km, and (**d**) 9 km. (**b**) is the same as Fig. 2**a**. (**e**) Distribution of earthquakes ($M \geq 1$) used to create **a-d**.

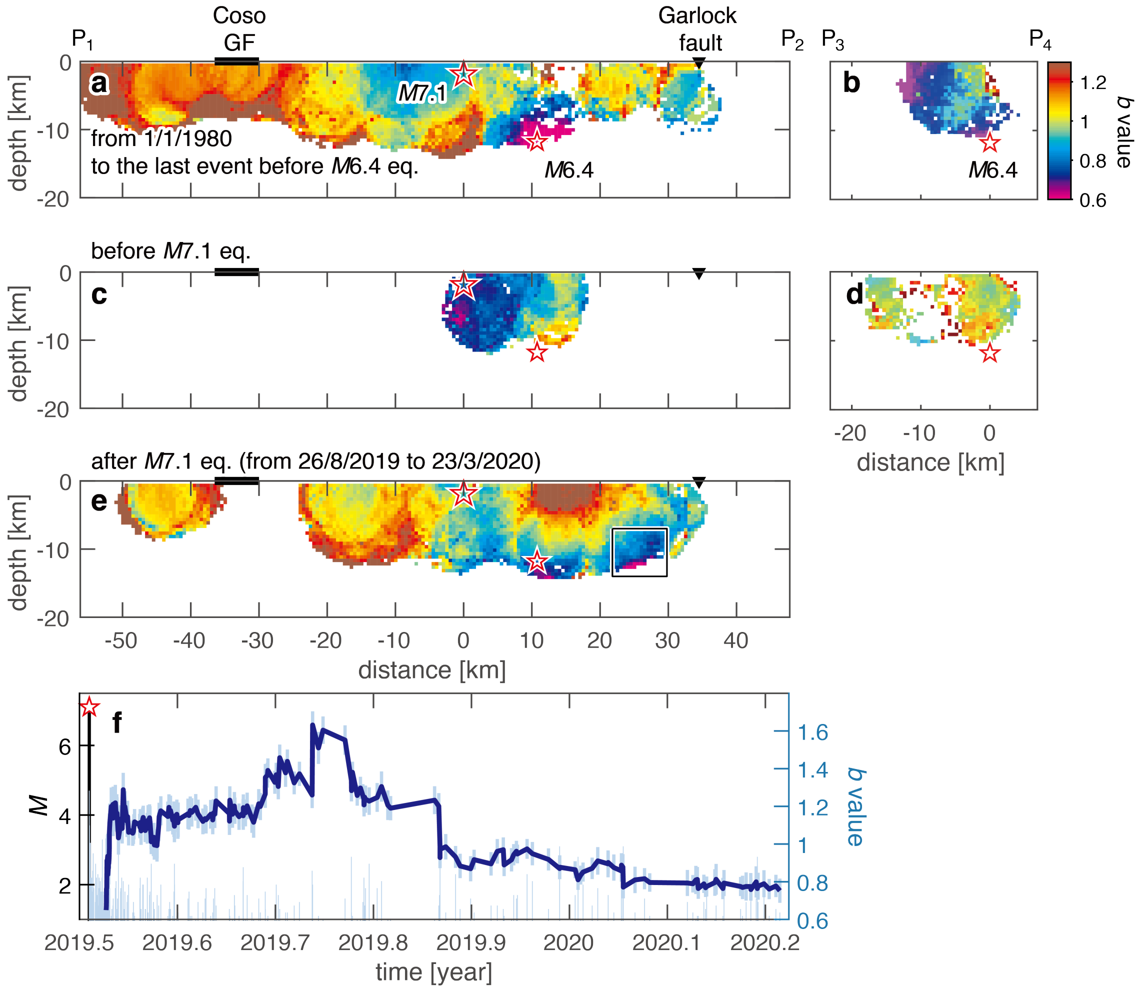

Supplementary Figure 4. Same as Fig. 2 for the case when M_c is increased by 0.2, where slip distribution same as that in Fig. 2**f** and frequency-magnitude distribution corresponding to that in Fig. 2**g** are not shown in this figure. Using the software package ZMAP[47], every local M_c was interactively increased by the prescribed value of 0.2. Then, *b*-values were computed for every node as performed in creating the *b*-value cross-sections and timeseries in Fig. 2. Earthquake data used to create the cross-sections in **a**-**e** and the timeseries in **f** are the same as those used for creating Fig. 2.

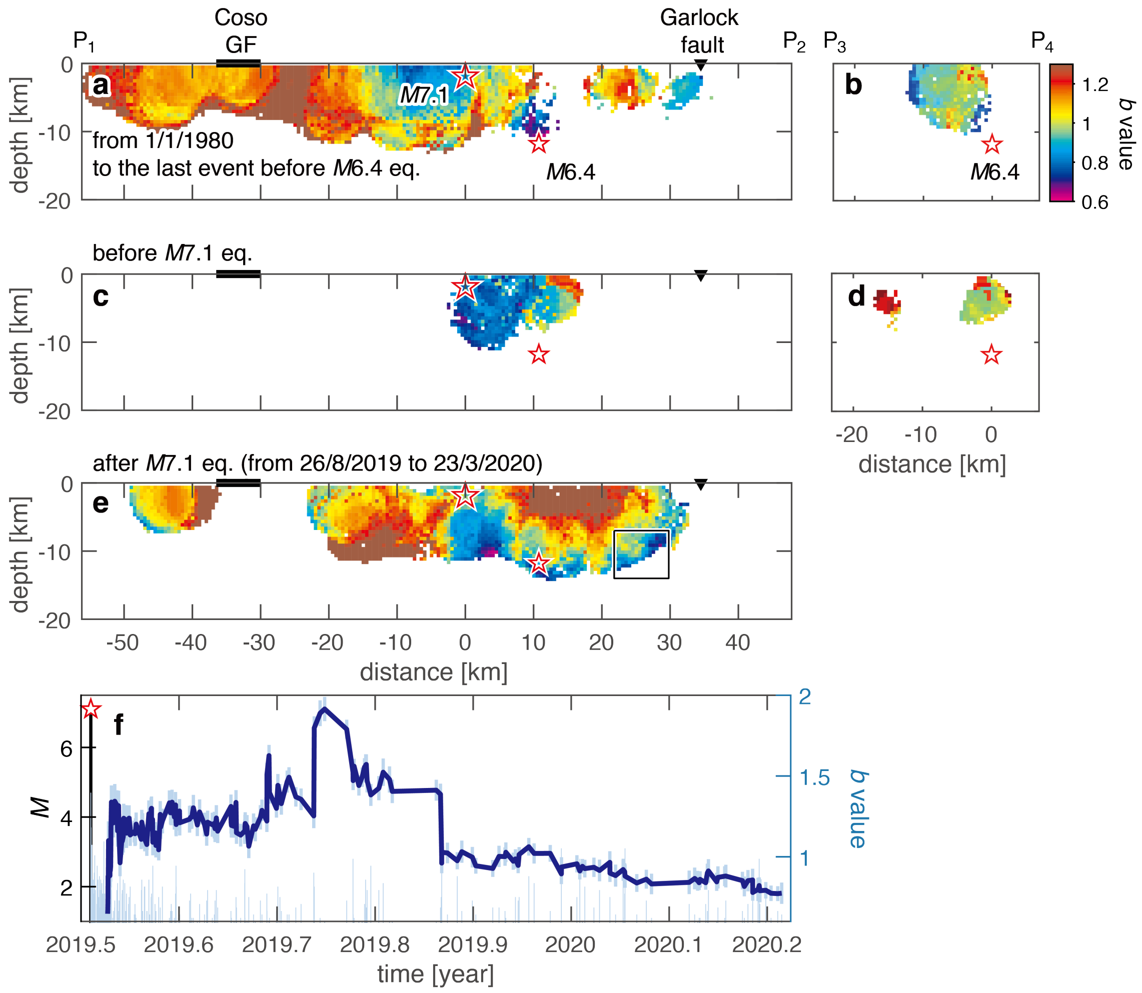

Supplementary Figure 5. Same as Supplementary Fig. 4 for the case when M_c is increased by 0.5.

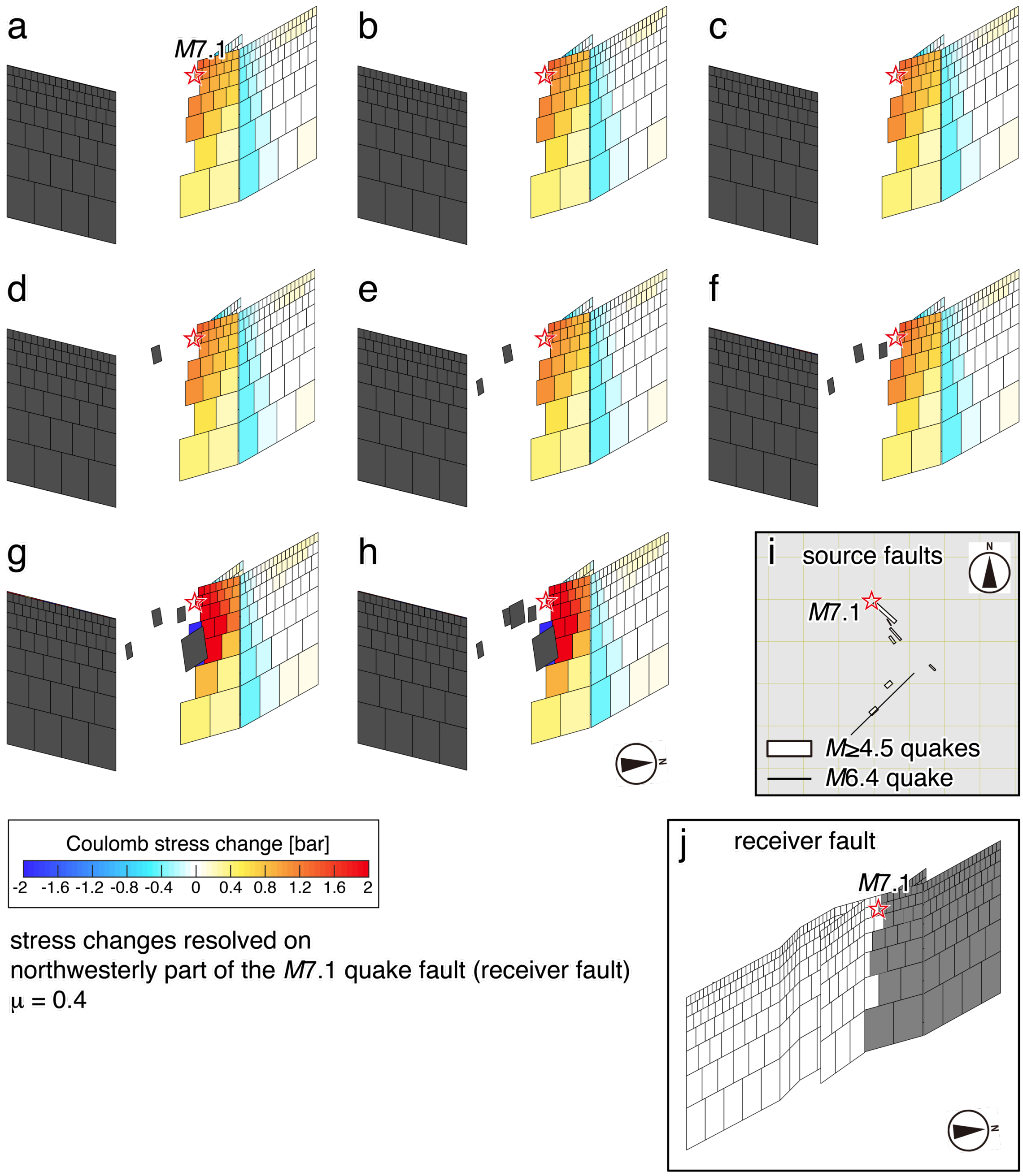

Supplementary Figure 6. Variation of changes in Coulomb stress across different source fault models. To show how Coulomb stress changes imparted by the *M*6.4 quake and the following *M*≥4.5 events played a role in triggering the eventual *M*7.1 quake, the number of source faults shown in **i** was increased over time from **a** to **h**. To easily see changes in Coulomb stress near the *M*7.1 hypocenter indicated by a star in response to an increase in the number of source faults, the northwesterly part of the fault ruptured by the *M*7.1 quake is shown. (**a**) Perspective view of distribution of the changes in

Coulomb stress as a result of the *M*6.4 quake (source fault in dark grey). (**b**) Same as **a** for a result of the *M*6.4 quake and the first event with *M*4.5. This event, whose fault was located behind the *M*6.4-quake fault, occurred 4 minutes after the *M*6.4 quake. (**c**) Same as **b** but the second event having *M*4.6 (1 hour and 8 minutes after the *M*6.4 quake) was added to source faults. This fault was again located behind the *M*6.4-quake fault. (**d**) Same as **c** but the third event with *M*4.6 (1 hour and 25 minutes after the *M*6.4 quake) was added to source faults. The same applies to **e**-**h**. (**a**, **h**) Same as the upper inset of Fig. 3**a** and Fig. 3**a**, respectively, but only the northwesterly part of the *M*7.1-quake fault are shown in **a** and **h**. (**i**) Source faults projected onto the Earth's surface. The *M*6.4-quake fault is indicated by a segment, because its dip is 90º, indicating a vertical fault. (**j**) Perspective view of the receiver *M*7.1-quake fault. Light-grey area of the receiver fault is the northwesterly part of the *M*7.1-quake fault, while white area is the southeasterly part of it. A preliminary calculation showed that the influence of $M<4.5$ events on changes in Coulomb stress was small and negligible.

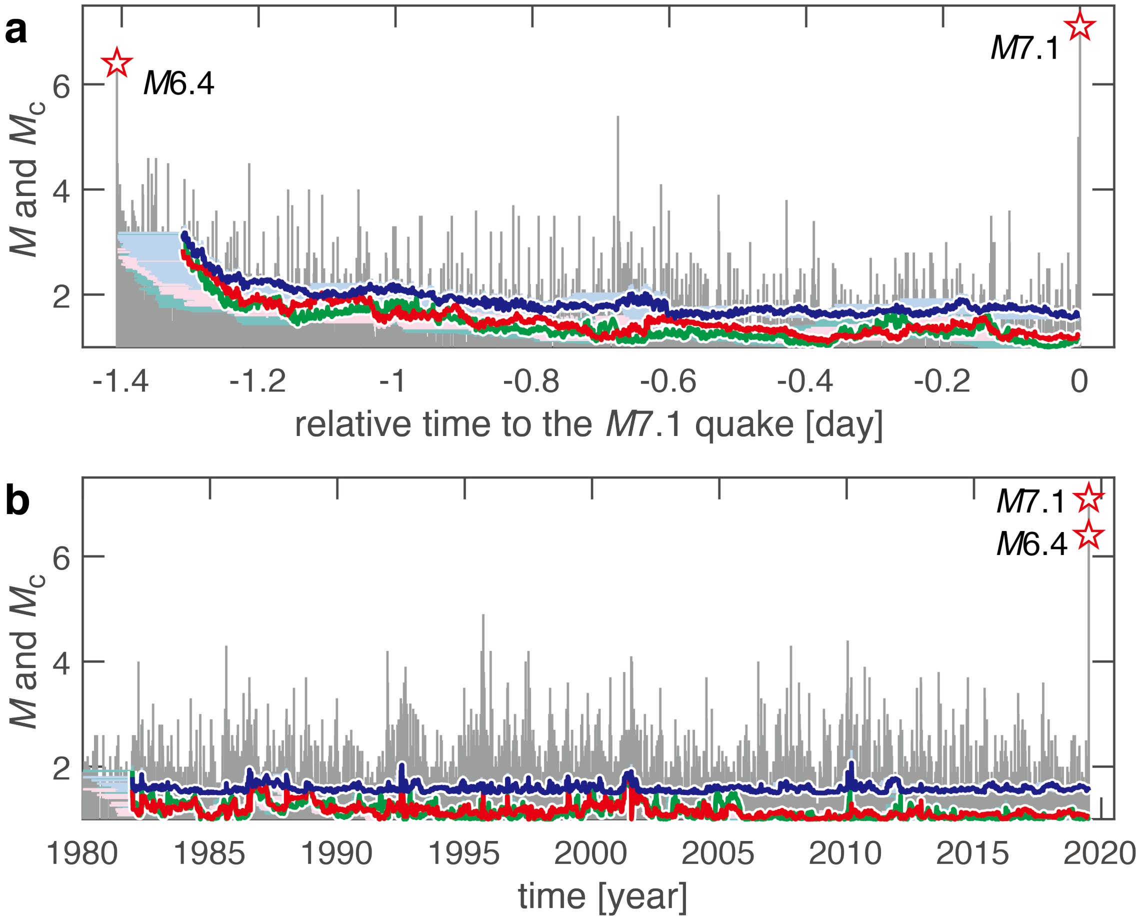

Supplementary Figure 7. Time-dependent M_c- and M-values. (**a**) Same as the inset of Fig. 2**c**, but including M_c estimated by using two other methods: the MAC method (red curve) and the GOF method (green curve). Note that the M5.4 quake, which is the largest event during the period between the M6.4 and M7.1 quakes, occurred at a relative time of -0.672 days. Seismicity from the first event after the M5.4 quake to the last event before the M7.1 quake was used to create Figs. 2**c** and 2**d** and Supplementary Figs. 4**c**, 4**d**, 5**c**, and 5**d**. (**b**) Same as **a** for seismicity along the rupture of the M7.1 quake (from P_1 to P_2) since 1 January 1980 until the last event before the M6.4 quake.

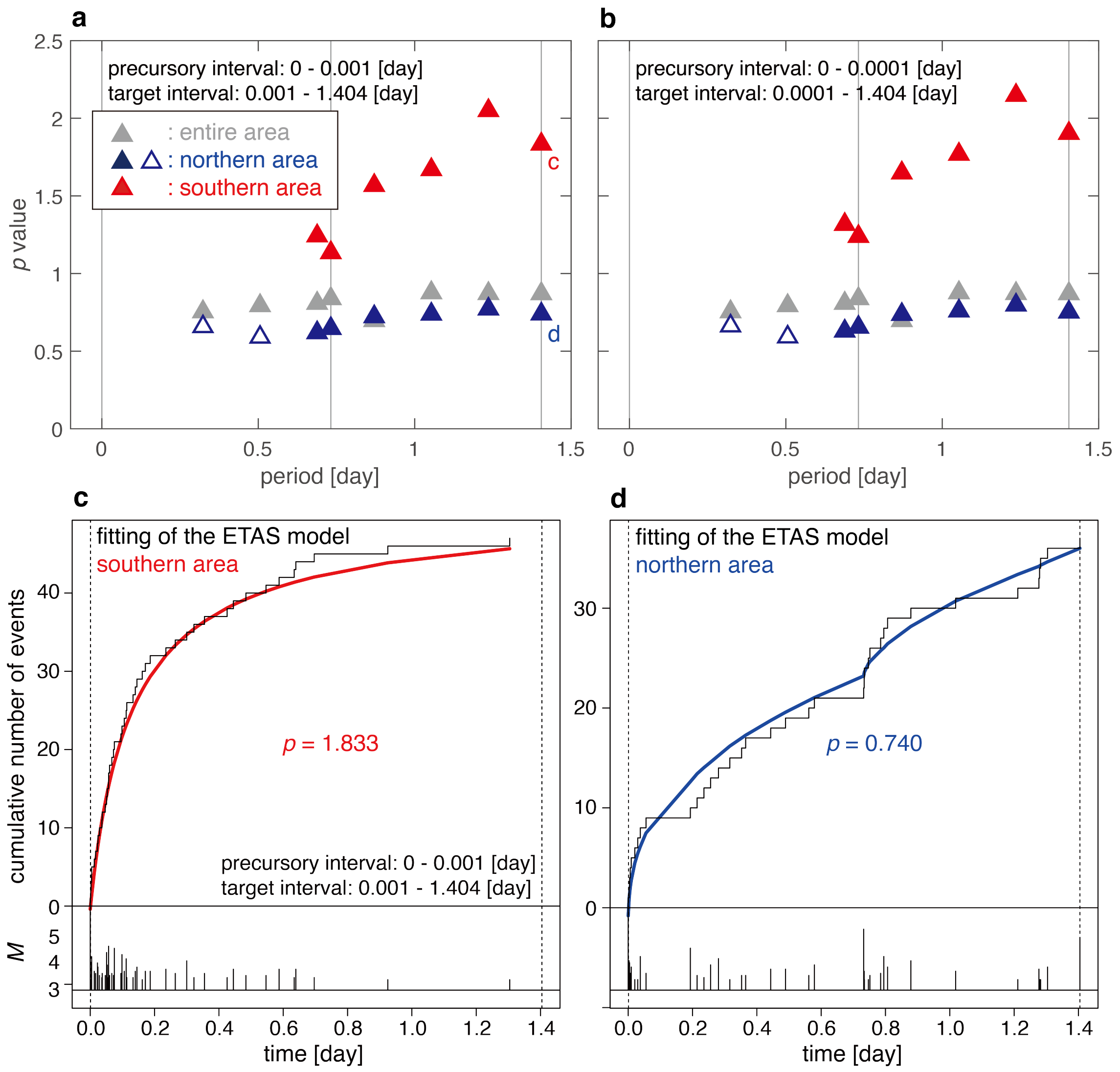

Supplementary Figure 8. ETAS model fitting. (**a**) Same as Fig. 3, but the ETAS model[55,56] was used. In **a**, data points, indicated by c and d, are the *p*-values obtained by fitting the ETAS model (red) to the accumulated number of $M \geq 3$ events in the southern area in **c** and the northern area in **d** during the period between the *M*6.4 and *M*7.1 quakes (corresponding to the analyzed period of 1.404 days). The interval between the dashed lines is the target interval for which the ETAS model parameters were computed[55,56]. The target interval started from 0.001 days since the *M*6.4 quake. Precursory interval ranges from the time of the *M*6.4 quake (0 days) to 0.001 days. Bottom panel in **c** and **d** shows a magnitude-time diagram for $M \geq 3$ events. (**b**) Same as **a** for the precursory interval of 0-0.0001 days and the target interval of 0.0001-1.404 days.

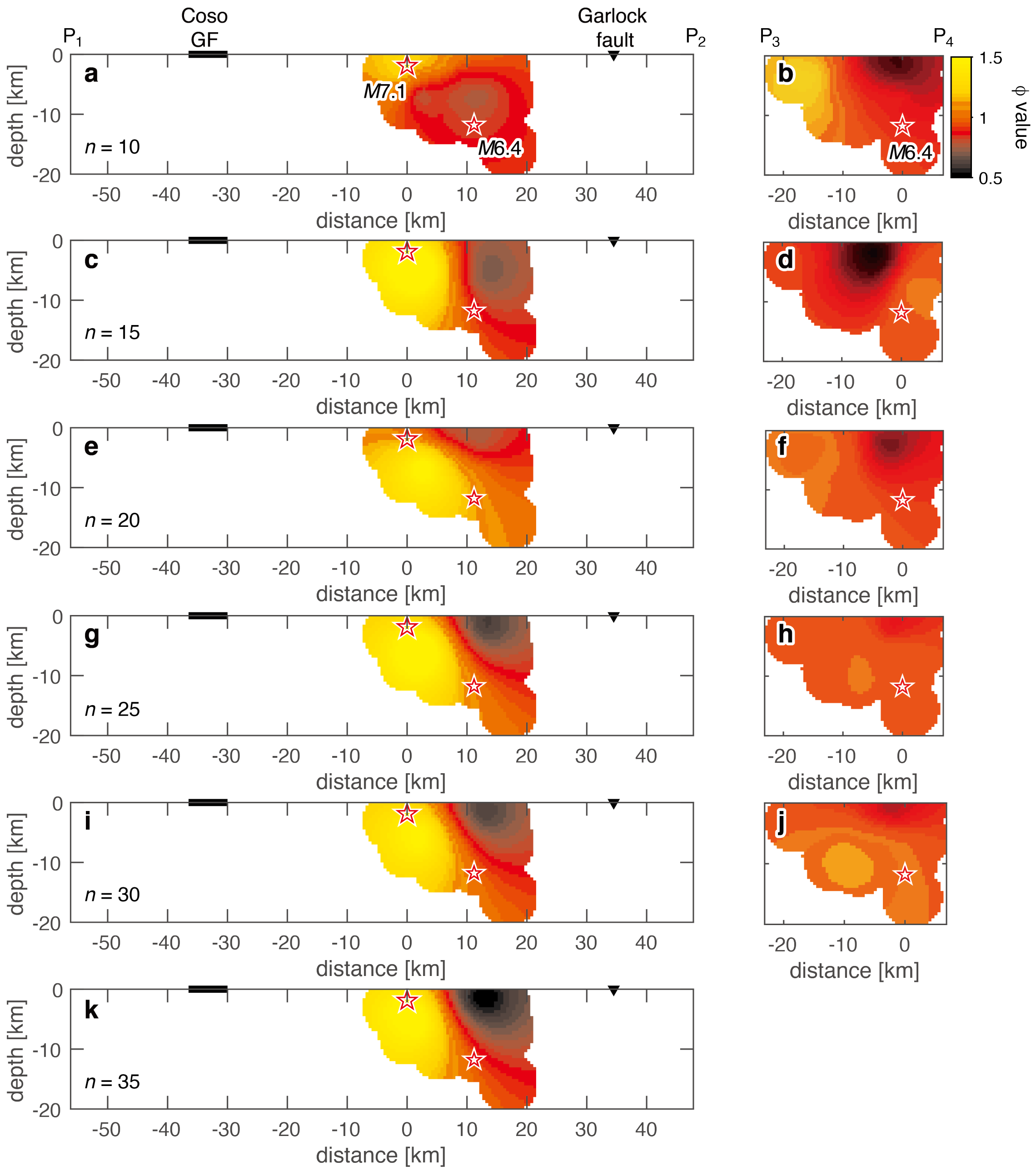

Supplementary Figure 9. Distributions of ϕ-values immediately before the *M*7.1 quake. ϕ-values was computed for seismicity ($M\geq3$) during the period between the *M*6.4 and *M*7.1 quakes along the fault ruptured by the *M*7.1 quake (left panels) and the fault ruptured by the *M*6.4 quake (right panels), using different *n*-values: (**a**, **b**) 10, (**c**, **d**) 15, (**e**, **f**) 20, (**g**, **h**) 25, (**i**, **j**) 30, and (**k**) 35. A distribution of ϕ-values using n=35 along the fault ruptured by the *M*6.4 quake was not created because the number of events ($M\geq3$) was less than $2n$ (=70).

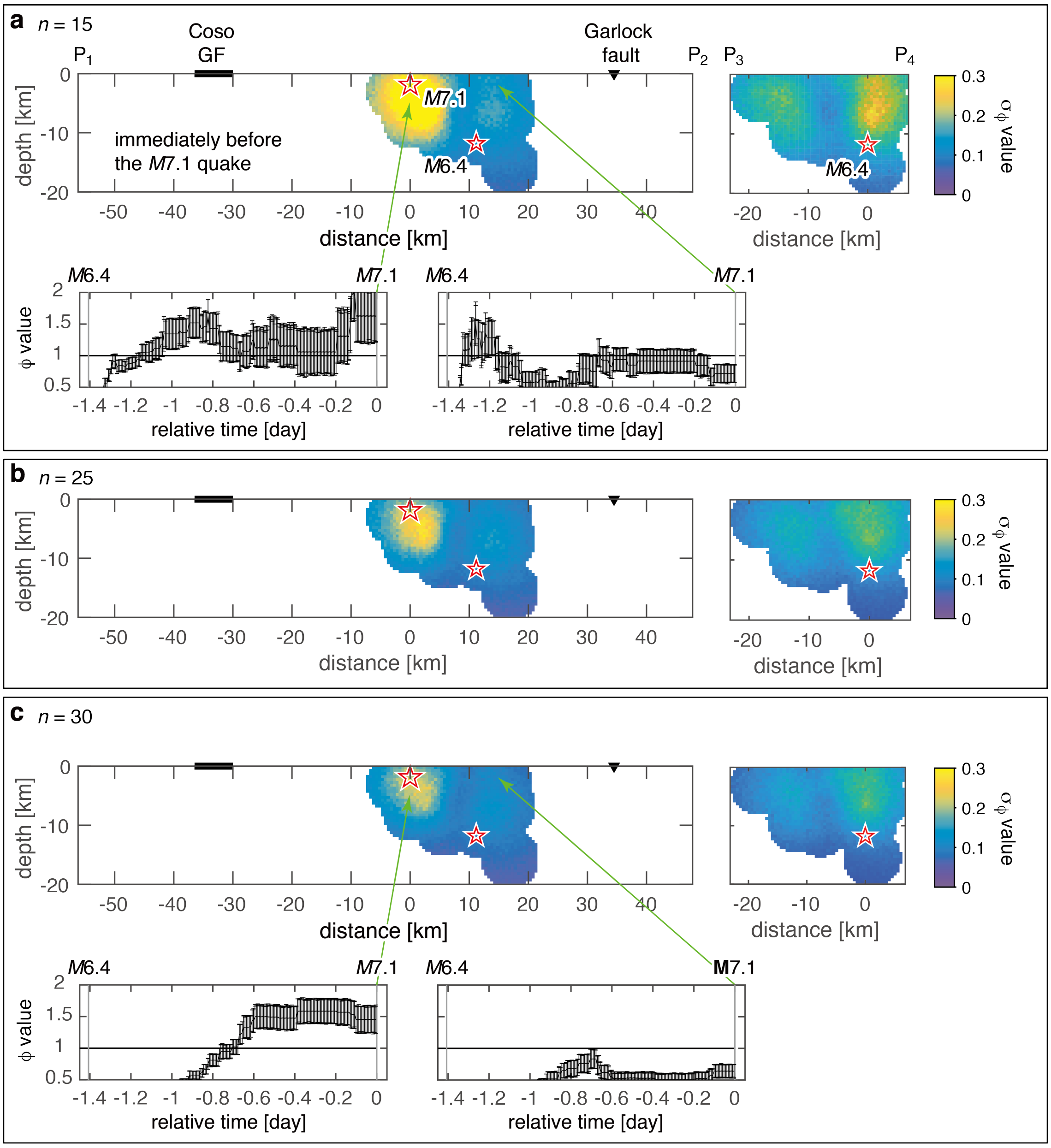

Supplementary Figure 10. Uncertainties of ϕ-values, σ_ϕ. (**a**) Distribution of σ_ϕ-values with n=15 at the time immediately before the *M*7.1 quake, created for seismicity (M≥3) during the period between the *M*6.4 and *M*7.1 quakes along the fault ruptured by the *M*7.1 quake (top left panel) and the fault ruptured by the *M*6.4 quake (top right panel). The corresponding distributions of ϕ-values are shown in Supplementary Figs. 9**c** and 9**d**. Bottom panels: plot of ϕ as a function of relative time to the *M*7.1 quake at the locations indicated by arrows. (**b**) Same as the top panels of **a** for n=25. The corresponding distributions of ϕ-values and the timeseries of ϕ-values are shown in Fig.

5. (**c**) Same as **a** for n=30. The corresponding distributions of ϕ-values are shown in Supplementary Figs. 9**i** and 9**j**.

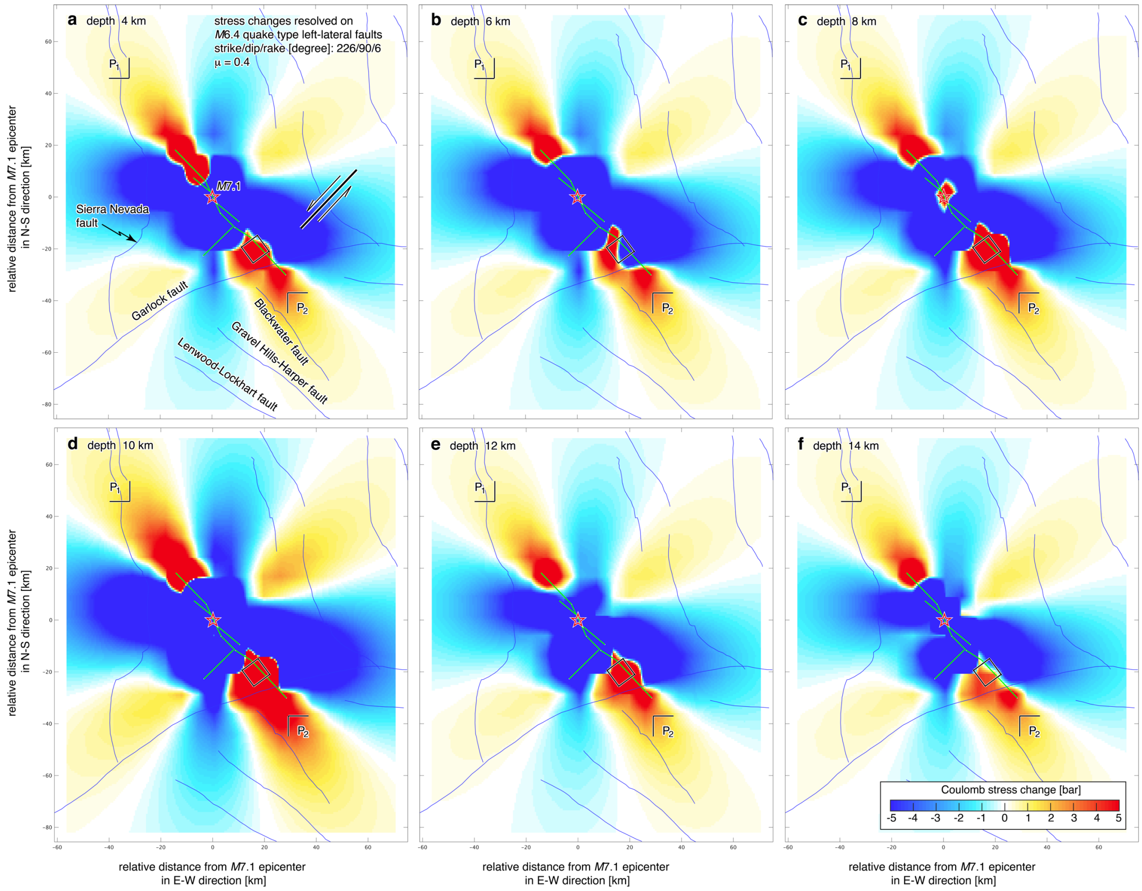

Supplementary Figure 11. Changes in Coulomb stress as a result of the *M*6.4 and *M*7.1 quakes (green segments). Stress changes resolved on *M*6.4-quake-type right-lateral faults (black line with a half-arrow pair) at a depth of (**a**) 4 km, (**b**) 6 km, (**c**) 8 km, (**d**) 10 km, (**e**) 12 km, and (**f**) 14 km. **d** is the same as the left panel of Fig. 3**b**. Star indicates the *M*7.1 hypocenter. Rectangle indicates an area of low *b*-values shown by the rectangle in Fig. 2**e** that displays the cross-section extending from P_1 to P_2.

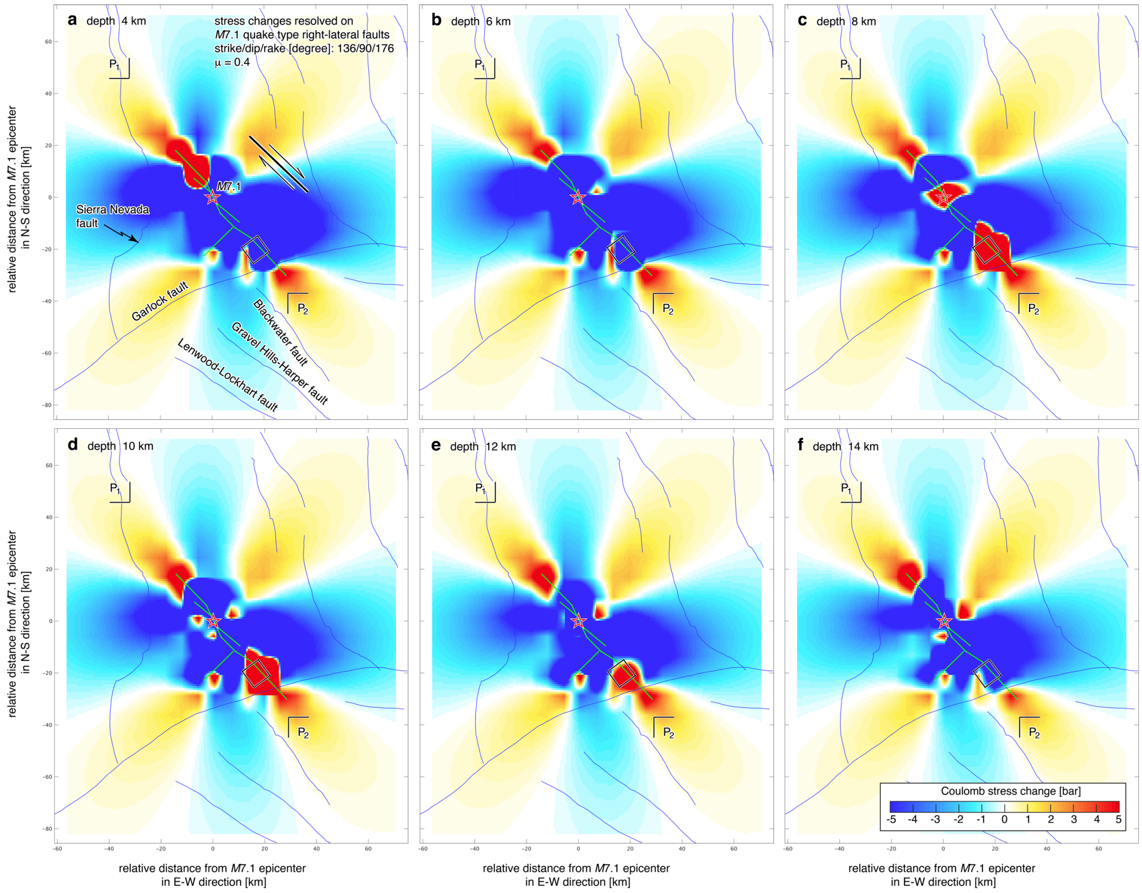

Supplementary Figure 12. Same as Supplementary Fig. 11 for stress changes resolved on *M*7.1-quake-type right-lateral faults (black line with a half-arrow pair). **d** is the same as the right panel of Fig. 3**b**.
